# Editorial: Systems pharmacology in the spotlight: trending technologies in network biology and drug discovery

**DOI:** 10.3389/fphar.2024.1535754

**Published:** 2024-12-10

**Authors:** Raghuveera Kumar Goel, Kiven Erique Lukong, Andrew Emili

**Affiliations:** ^1^ Division of Oncological Sciences, Knight Cancer Institute, Oregon Health and Science University (OHSU), Portland, OR, United States; ^2^ Department of Biochemistry, Microbiology and Immunology, College of Medicine, University of Saskatchewan, Saskatoon, SK, Canada

**Keywords:** systems pharmacology, systems biology, protein-protein interaction (PPI), proteomics and bioinformatics, network biology, high throughput screen (HTS), drug, therapy

## Introduction

Systems pharmacology is an interdisciplinary field that combines pharmacological insights with systems biology to better understand the complex interactions between drugs, biological systems, and diseases. It moves beyond traditional pharmacology by integrating computational modeling, network analysis, and high-throughput data to predict drug effects at the systems level, including therapeutic benefits and potential side effects. By considering the dynamic interplay of molecular pathways, cellular responses, and organism-level physiology, systems pharmacology provides a holistic framework for drug discovery, personalized medicine, and the optimization of treatment strategies.

Recent advancements in systems pharmacology are deepening our understanding of complex disease mechanisms and have facilitated the development of targeted therapeutic interventions. Some of these engaging paradigms in early-stage discovery efforts involving kinase-inhibition for example, have been extensively reviewed by Stephenson and Higgins. In this editorial, we spotlight the authors’ contributions to our special Research Topic on systems pharmacology, emphasizing the importance of diverse methodologies. These include high-throughput screening, *in silico* modeling of drug-ligand docking with network-level interactions, and experimental validations (both *in vitro* and *in vivo*), all aimed at elucidating effects on cellular signaling networks within a therapeutic context.

## High-throughput screening approaches

### Anti-pancreatic cancer activity of epimedium herb

Chen et al. explore the Epimedium herb’s therapeutic potential against pancreatic cancer. Through high-performance liquid chromatography, network pharmacology, and molecular docking, the authors identified active compounds like icariin and baohuoside I, targeting pathways such as Interleukin-4 and interleukin-13 signaling. *In vitro* assays demonstrated that Epimedium extracts significantly reduced Panc-1 cell viability, suggesting its promise as a multi-targeted therapeutic agent for pancreatic cancer.

### Integrated metabolomics and proteomics in end-stage kidney disease

Lin et al. employed an integrative approach combining metabolomics and proteomics to identify biomarkers associated with hemodialysis in end-stage kidney disease (ESKD). Their analysis uncovered significant alterations in metabolic and protein profiles, providing insights into the physiological changes induced by hemodialysis. These findings have the potential to guide the development of targeted therapies and improve patient outcomes in ESKD management.

## *In silico* analyses and experimental validation

### Mechanisms of action of Sappan Lignum for prostate cancer treatment

Li et al. investigated the antitumor properties of Sappan Lignum, derived from *Caesalpinia sappan L.*, against prostate cancer (PCa). The study identified 21 major active compounds within Sappan Lignum and their 32 highly probable target proteins mapped from 821 differentially expressed genes associated with PCa. Through network pharmacology and molecular docking, they pinpointed eight key targets, notably BCL-2 and CCNB1, with the p53 signaling pathway being central to the herb’s antitumor effects. *In vitro* experiments demonstrated that 3-deoxysappanchalcone (3-DSC), a principal component, inhibited PCa cell proliferation, migration, and induced apoptosis, primarily by modulating the p53/p21/CDC2/CCNB1 pathway. These findings underscore Sappan lignum’s promise as a multi-targeted therapeutic agent for PCa.

### Luoshi Neiyi prescription in endometriosis: a network pharmacology approach

Wu et al.’s study explores the underlying mechanisms of action of Luoshi Neiyi prescription (LSNYP) for treating endometriosis, utilizing serum pharmacochemistry and network pharmacology approaches. By identifying active components and their targets, the research highlights LSNYP’s regulation of hypoxia and inflammation-related pathways, notably the HIF1A/EZH2/ANTXR2 axis. Experimental validation demonstrated that LSNYP modulates key cellular proteins, suggesting its therapeutic potential in endometriosis management. This integrative approach provides a scientific basis for the therapeutic effects of LNP in endometriosis management.

### Long Mu Qing Xin Mixture for ADHD: a multi-methodological study

Li et al. investigated the formulated medicine, Long Mu Qing Xin Mixture (LMQXM) for treating attention deficit hyperactivity disorder (ADHD). Network pharmacology and molecular docking approaches identified key components; beta-sitosterol, stigmasterol, rhynchophylline, baicalein, and formononetin, that interact with dopamine receptors DRD1 and DRD2. *In vivo* experiments confirmed that LMQXM modulates the dopamine and cAMP signaling pathways, suggesting its therapeutic potential for ADHD.

### Systems biology approach to glioblastoma multiforme

This study (Alqahtani et al.) highlighted matrix metallopeptidase 9 (MMP9) as a critical molecular target in glioblastoma multiforme (GBM) pathophysiology. By prioritizing molecular compounds sourced from drug-ligand interaction databases and molecular docking, carmustine, lomustine, marimastat, and temozolomide demonstrated significant binding affinities to MMP9, suggesting their utility in modulating GBM’s molecular network for enhanced therapeutic outcomes.

### Desert flora in cancer therapy: network pharmacology and molecular modeling

Alblihy systematically explored the anticancer potential of phytochemical compounds derived from Arabian flora using network pharmacology and molecular modeling. The work highlighted multiple pathways through which these natural compounds exert their effects, including apoptosis, cell cycle arrest, and inhibition of metastasis. The study emphasizes the importance of integrating traditional knowledge with modern computational techniques to discover novel therapeutic agents in ovarian cancer.

### GHRP-6 mitigates doxorubicin-induced cardiotoxicity

Berlanga-Acosta et al. explored the cardioprotective effects of Growth Hormone Releasing Peptide-6 (GHRP-6) against doxorubicin (Dox)-induced cardiotoxicity. Through echocardiography, histopathology, and molecular analyses, the study found that GHRP-6 administration alongside Dox prevented myocardial fiber degradation and ventricular dilation, thereby preserving left ventricular systolic function. Mechanistically, GHRP-6 enhanced antioxidant defenses, upregulated the pro-survival gene Bcl-2, and maintained mitochondrial integrity in cardiomyocytes. These findings suggest that GHRP-6 activates pro-survival mechanisms, offering a potential therapeutic strategy to mitigate Dox-induced cardiac damage.

Collectively, these studies underscore four key tools of systems pharmacology for unraveling complex disease mechanisms and identifying potential therapeutic agents: high-throughput screening, biomolecular network modeling, simulated structural docking, and experimental validation. By integrating computational and experimental approaches, researchers can accelerate the discovery of novel treatments and deepen our understanding of disease pathology.

## Looking forward; the age of generative artificial intelligence (AI)

The future of generative AI within systems pharmacology is poised to be transformative. Generative AI holds the potential to automate the design of therapeutic molecules and simulate their interactions across diverse biological systems, significantly reducing the time and cost required to move from concept to clinical trials. By modeling vast chemical spaces in predicting drug-target interactions and synergizing systems-level data such as multi-omics and dynamic network analyses, generative AI can enhance drug optimizations as well as evaluate safety and efficacy through iterative simulations on virtual patients. By democratizing drug discovery and fostering interdisciplinary collaboration, generative AI promises a more efficient and sustainable future for systems pharmacology.

